# The Impact of a Web-Based App (eBalance) in Promoting Healthy Lifestyles: Randomized Controlled Trial

**DOI:** 10.2196/jmir.3682

**Published:** 2015-03-02

**Authors:** Jenny Safran Naimark, Zecharia Madar, Danit R Shahar

**Affiliations:** ^1^Robert H. Smith Faculty of Agriculture, Food and EnvironmentHebrew University of JerusalemRehovotIsrael; ^2^S. Daniel Abraham International Center for Health and Nutrition,Department of Public Health, Faculty of Health SciencesBen-Gurion University of the NegevBeer ShevaIsrael

**Keywords:** Web-based, healthy lifestyle, mobile apps, mhealth

## Abstract

**Background:**

The use of Web-based apps to promote a healthy lifestyle is increasing, although most of these programs were not assessed using suitable epidemiological methods. We evaluated the effectiveness of a newly developed Web-based app in promoting a healthy lifestyle and educating adults on such lifestyles. We also analyzed predictors for success in acquiring and maintaining a healthy lifestyle.

**Objective:**

Our aim was to compare people receiving a new Web-based app with people who got an introductory lecture alone on healthy lifestyle, weight change, nutritional knowledge, and physical activity, and to identify predictors of success for maintaining a healthy lifestyle.

**Methods:**

Subjects were recruited from the community and were randomized into intervention and control groups. The intervention subjects received access to the app without any face-to-face support; the control subjects continued their standard lifestyle. Measurements were taken by the researcher at baseline and after 14 weeks and included weight and waist circumference. Nutritional knowledge, diet quality, and physical activity duration were obtained using online questionnaires. The new Web-based app was developed based on current US Department of Agriculture and Israel Ministry of Health recommendations for healthy lifestyle. The app provides tools for monitoring diet and physical activity while instructing and encouraging healthy diet and physical activity.

**Results:**

Out of 99 subjects who were randomized into app and control groups, 85 participants (86%) completed the study, 56 in the intervention and 29 in the control group. The mean age was 47.9 (SD 12.3) years, and mean Body Mass Index was 26.2 (SD 3.9). Among the intervention group only, frequency of app use was 2.7 (SD 1.9) days/week. The mean change in physical activity was 63 (SD 20.8) minutes in the app group and -30 (SD 27.5) minutes in the control group (*P*=.02). The mean weight change was -1.44 (SD 0.4) kg in the app group and -0.128 (SD 0.36) kg in the control group (*P*=.03). Knowledge score increased significantly in the app group, 76 (SD 7.5) to 79 (SD 8.7) at the end of the study (*P*=.04) compared with the control group. Diet quality score also increased significantly at the end of the study, from 67 (SD 9.8) to 71 (SD 7.6; *P*<.001) in contrast to the control group. Success score (represents the success in maintaining healthy lifestyle) was higher among the app group (68%) compared with 36% in the control group (*P*<.001). The app frequency of use was significantly related to a higher success score (*P*<.001).

**Conclusions:**

We showed a positive impact of a newly developed Web-based app on lifestyle indicators during an intervention of 14 weeks. These results are promising in the app’s potential to promote a healthy lifestyle, although larger and longer duration studies are needed to achieve more definitive conclusions.

**Trial Registration:**

Clinicaltrial.gov number: NCT01913496; http://www.clinicaltrials.gov/ct2/show/NCT01913496 (Archived by WebCite at http://www.webcitation.org/6WSTUEPuJ).

## Introduction

Although chronic diseases have reached epidemic proportions, they could be significantly reduced through prevention or reduction of their risk factors, early detection, and timely treatments [1]. Most chronic diseases are strongly associated and causally linked with four particular behaviors: tobacco use, physical inactivity, unhealthy diet, and excessive consumption of alcohol. These behaviors lead to four key metabolic/physiological changes: hypertension, overweight/obesity, hyperglycemia, and hyperlipidemia. In terms of attributable deaths, the leading chronic disease risk factors globally are hypertension (attributed with 13% of global deaths), tobacco use (9%), hyperglycemia (6%), physical inactivity (6%), and overweight and obesity (5%) [2].

A healthy lifestyle was proven to lower the risk for chronic diseases. In the European Prospective Investigation into Cancer and Nutrition (EPIC) study, 23,153 German participants aged 35-65 were followed for a mean of 7.8 years. Adherence to four health behaviors not smoking, exercising 3.5 hours per week, eating a healthy diet (high intake of fruits, vegetables, and whole-grain bread, and low meat consumption), and having a Body Mass Index (BMI) <30 kg/m^2^ at baseline was associated with 78% lower risk for developing chronic disease (diabetes 93%, myocardial infarction 81%, stroke 50%, and cancer 36%) than participants without these protective factors [3,4].

Despite the importance of following healthy lifestyle recommendations for preventing or lowering the incidence of chronic diseases, multiple population studies showed that only a minority of individuals adheres to healthy lifestyle behaviors. In a comparative analysis of middle-aged adults aged 40-74 years participating in the National Health and Nutrition Examination Study (NHANES III) 1988-1994 and 2001-2006 surveys, the proportion of adults who adhered to all five healthy habits (at least five fruits and vegetables/day, regular exercise >12 times/month, maintaining a BMI between 18.5 and 29.9 kg/m^2^, moderate alcohol consumption, and not smoking) decreased from 15% to 8% [5,6]. Additional available data suggest that 31% of the world’s population is not meeting the minimum recommendations for physical activity [7].

The number of behavior change interventions that have become available through the Internet has increased steadily over the last decade [8]. Evidence exists to support the effectiveness of Web-based interventions in changing behaviors. Research has shown these interventions to be effective in different areas of health care [9-14]. Among them are promoting physical activity [15], nonsmoking behavior [10], and weight loss [16]. However, the effects are usually small [17]; the actual reach of Internet-delivered interventions seems lower than expected and attrition rates are generally high [10]. To the best of our knowledge, there have not been any randomized controlled trials (RCTs) of a Web/mobile phone app as a healthy lifestyle intervention in itself that focuses on education and self-monitoring of diet and physical activity for the healthy population.

A trial of this type is important because Web/mobile phone apps are readily available to the public and provide an opportunity to develop interventions with lower costs, less burden, and a greater reach, particularly as mobile phone use rates continue to rise. Adding Web/mobile phone technology offers a significant potential for improving the user’s self-monitoring experience and adherence to health promotion programs and thus to enjoy their benefits.

In this study, we hypothesized that using a theory-guided, technology-supported, healthy lifestyle–promoting Web-based app will result in high use, improved nutritional knowledge, increased duration of physical activity, and greater weight loss compared to a control approach.

The objective of the study was to compare people receiving a new Web-based app with people who got an introductory lecture on healthy lifestyle alone, on resulting weight change, nutritional knowledge, and physical activity, and to identify predictors of success for maintaining a healthy lifestyle.

## Methods

### Eligibility and Study Design

People from the community (high-tech companies, kibbutzim, large organizations) were recruited between the end of 2012 and January 2013 from the south and center of Israel, via an email invitation. The invitation described the research goals and informed the participants that their participation was voluntary and involved no incentive payments or gifts.

The study lasted 14 weeks and had two arms: intervention (Web-based app) and control. Eligible participants were healthy people, aged ≥18 years with Web and email experience, interested in a healthy lifestyle, and willing to commit the necessary time and effort to the study. Exclusion criteria were participating in a program for weight reduction, pregnancy, and lactation.

The first face-to-face meeting of the study was the introductory meeting. In this meeting, all participants received a presentation on a healthy lifestyle, recommendations for healthy nutrition, and the clinical benefits of physical activity for the recommended weekly duration. The participants signed an informed consent form and were randomized to intervention and control groups.

The members of the app group received a password for the app and were instructed to use it as much as they wanted to. The members of the control group were instructed to continue living a healthy lifestyle as they understood it and based on the presentation in which they participated. The control group did not receive any other intervention.

Anthropometric measurements of weight and waist were obtained by the researcher in the introductory meeting. Nutrition knowledge, diet quality, and physical activity type and duration questionnaires were completed by participants through the Internet (Google docs). Anthropometric measurements and questionnaires were repeated after 14 weeks (the last face-to-face meeting). The intervention participants also completed a satisfaction questionnaire at the end of the study. In total there were only two face-to-face meetings between the researcher and the study participants during the study period.

### Randomization

Following the introductory meeting, and after signing an informed consent form, participants were randomized to the intervention and control groups. The randomization was performed in clusters according to the place of recruitment: kibbutz, company, organization. Participants were numbered using a random number table where the numbers were randomly assigned to intervention and control groups using a 2:1 ratio.

This ratio was used in order to enhance recruitment by increasing the chances of being in the intervention group. This ratio enabled us to recruit more people and to achieve our calculated sample size.

### Ethics and Informed Consent

Ethics approval was received by the Hebrew University, Faculty of Agriculture Food and Environment (AGHS-07.12). Each participant provided written informed consent at the beginning of the study. This study is a registered clinical trial (NCT01913496).

### Intervention

**Figure 1:**
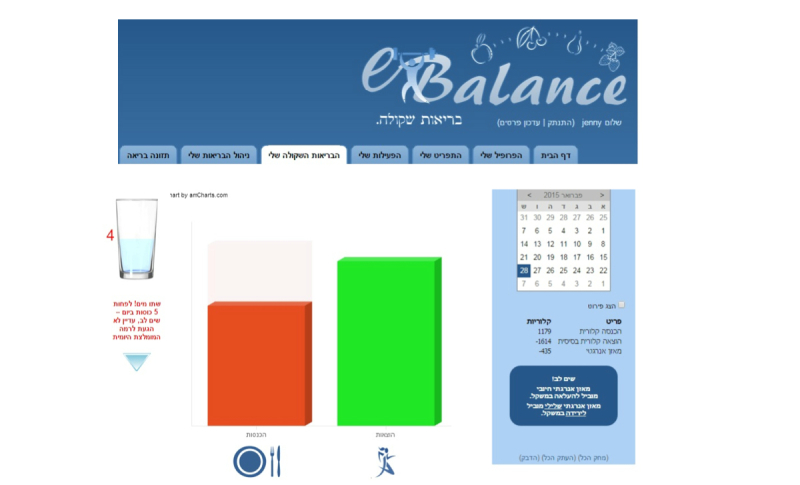
Screenshot of the eBalance Web-based app.

**Figure 2:**
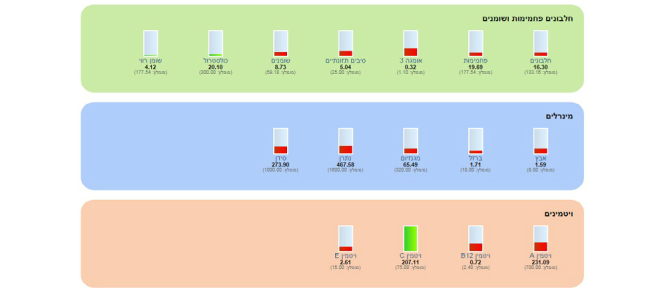
Monitoring key nutrients by the app. Nutrients are divided into three groups: (1) carbohydrates, proteins, fats, (2) minerals, and (3) vitamins (green bar – intake according to the recommendations, red bar – intake below the recommendations).

The new Web-based eBalance app was designed for a healthy non-professional audience interested in self-management and achievement of a healthy lifestyle. It was developed over a period of 1 year by one of the authors (JSN) and was programmed by a software expert (Figures 1 and 2). The app is based on guidelines published by the Israel Ministry of Health [18] and the Dietary Guidelines for Americans 2010 [19] recommendations for a Healthy Lifestyle. The theoretical basis of the app and the elements required to keep users engaged was given much consideration. The app was designed to be user-friendly for all types of populations, and specifically for people with only basic computer and Internet skills. If additional explanations are necessary, the user can watch videos with instructions on how to use the app (located in the app itself). eBalance was designed to be a helpful tool for facilitating self-management of a healthy lifestyle. Much thought was invested in enabling the easiest and fastest possible entry of data (food intake and physical activity).

The app enables the users to monitor their dietary intake and physical activity by receiving real-time feedback. Based on the Control Systems Theory (CST) of self-regulation [20], we postulated that the experience of receiving consistent and immediate feedback from eBalance would reinforce adherence to self-monitoring and healthy behavior change.

The essence of the app is based on the flexibility of choosing your own preferred diet within the Dietary Reference Intake (DRI) recommendations that are related to the user. This was achieved through feedback from the app. This feedback includes information on nutrient intake compared with the recommended level of DRI and potential food sources for the nutrients, in addition to monitoring the calorie intake and expenditure.

For example, Figure 1 shows the diet of a user who did not achieve the daily recommendations of several nutrients. In this case, the app can suggest which types of foods the user can add to their menu in order to reach the recommended level.

### Outcomes Assessment

#### App Usability

App usability was determined by frequency and convenience of use. Frequency of use was evaluated from the app database according to the reports of the users. Google Analytics was also used as an objective tool for evaluating visit duration and number of pages per visit. Convenience of use was reported by the app users via a satisfaction questionnaire. The satisfaction questionnaire was developed for eBalance based on a questionnaire developed by Shahar et al [21].

#### Lifestyle Questionnaires

The lifestyle questionnaires were adapted for use in the current study.

##### Nutrition Knowledge

There are two ways to improve knowledge using the app. The first is by entering the “Healthy Nutrition” page. This page contains information on the importance of the key nutrients for a healthy lifestyle and displays the food sources for these key nutrients. The second is through routine use of the app, which leads to learning about the caloric value of food intake or physical activity expenditure and also about the food’s key components.

Knowledge was assessed using a 43-item online self-report questionnaire based on Parmenter’s general nutrition knowledge questionnaire for adults [22].

##### Diet Quality

The diet quality questionnaire is a 16-item questionnaire based on Parmenter’s general nutrition knowledge questionnaire for adults [22].

##### Physical Activity

This questionnaire included 28 questions on the type, frequency, and duration of time each participant spent in physical activity per week. This questionnaire is based on an international physical activity questionnaire [23,24].

### Anthropometric Measurements

Weight was measured in kilograms with a portable digital scale (Beurer GmbH & Co. KG, Germany). Weight measurement was performed in light clothes and without shoes to the nearest 0.1 kg. Waist circumference was measured on the navel.

### Sample Size Calculation

The sample size for the RCT was calculated based on the findings from a previous pilot study. Cholesterol intake was used for the calculation; the sample size was calculated using *P=*.05, power=80%. A sample size of 50 participants in the app users group was found to be sufficient for detecting a 10% reduction in level of cholesterol intake at the end of study period compared with the baseline. We added 20% to the number of participants to compensate for potential dropouts.

### Statistical Analysis

Analyses were carried out using SPSS 21.0. Descriptive statistics were used to describe and compare baseline characteristics of participants in the app and control groups. Univariate analyses were performed first to compare pre- and post- values using the paired *t* test. The change from baseline was compared using the *t* test for independent samples. Categorical measurements were compared using the chi-square test. An analysis of variance (ANOVA) model followed by Scheffe post-hoc tests were used to compare differences between three groups (control and the two app groups, light users and heavy users). This analysis was performed post hoc and is presented separately from the main outcomes in each table and figure.

In order to estimate the effect of the intervention on lifestyle, we defined an integrated score (success score) based on the following parameters: decrease or maintain body weight, duration of physical activity equal to or above 150 minutes per week, and scores of at least 70 in knowledge and diet quality questionnaires. Each parameter contributed one point to the score, summing the 4 points for all users. An analysis of covariance (ANCOVA) model was applied to identify the factors that affect the score. Age and gender were included as confounders, frequency of use was entered as the tested factor with three levels: control (no use at all), light users (less than three usage days per week), and heavy users (at least three usage days per week). The model was also tested for app users versus the control group. The statistical significance level was defined as alpha*=*.05.

## Results

### Recruitment

**Figure 3:**
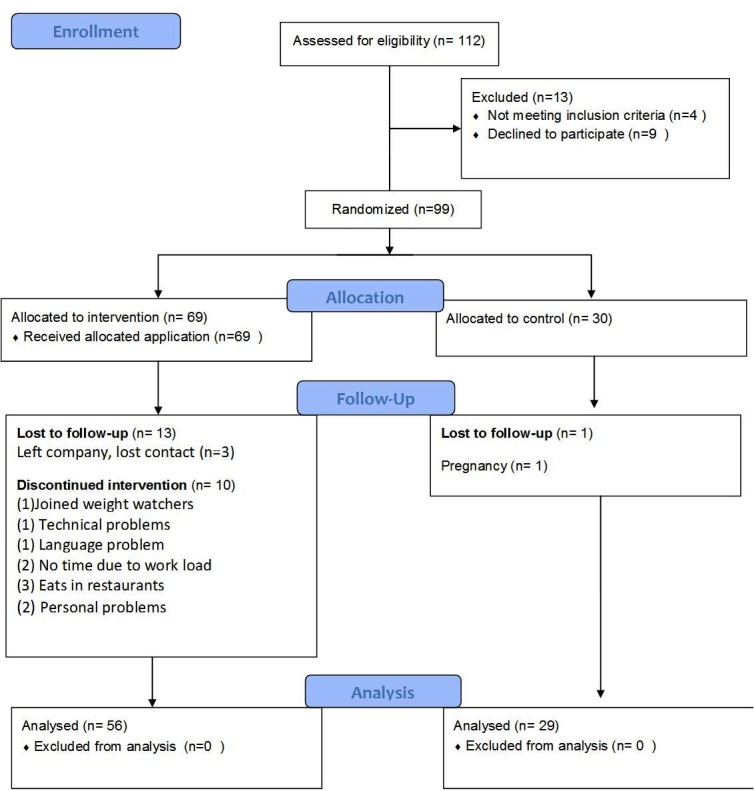
Flow of the participants through the RCT.

Of the 112 who agreed to participate in the study, 99 participants were found eligible and were randomized into two groups (Figure 3). A total of 85 participants completed the follow-up visit (86% retention rate); 56 of the 69 participants who started the study in the app group completed the follow-up (81% retention rate) and 29 of 30 participants who started the study in the control group completed the follow-up (97% retention rate). Three participants left their company and their contact was lost. One joined WeightWatchers, one had technical problems, one had a language problem, two had no time due to work load, three frequently ate in restaurants and did not always know the components of the food, and two had personal problems. In the control group, one participant did not complete the follow-up due to pregnancy. There were no differences in the demographic characteristics at baseline of the app non-completers group and the app users group.

### Baseline Data

Characteristics of the study participants are presented in Table 1. Our study included 85 adults: 54 (64%) women, 31 (36%) men, with a mean age of 47.9 years (SD 12.3). No differences in age, weight, BMI, height, and waist circumference existed between the app and control groups at baseline.

**Table 1:** Baseline characteristics of the RCT participants.

Characteristics	All (n=85)	Web-based app (n=56)	Control (n=29)	*P*
Age in years, mean (SD)	47.9 (12.3)	48.5 (11.3)	46.7 (14.2)	.53
Females, n (%)	54 (64)	33 (59)	21 (72)	.25
Weight in kg, mean (SD)	74.0 (15.4)	76.3 (15.1)	69.7 (15.2)	.07
Height in cm, mean (SD)	1.69 (0.1)	1.70 (0.1)	1.67 (0.1)	.09
BMI, mean (SD)	25.8 (4.0)	26.2 (3.9)	25.0 (4.4)	.19
Waist circumference in cm, mean (SD)	93.5 (12.4)	94.3 (11.7)	91.7 (14)	.39

### Frequency of Use

The mean frequency of use among the intervention group was 2.7 (SD 1.9) days a week (95% CI 2.2-3.2). The average period of use was 7.8 (SD 4.3) weeks; this is the period that a user stayed connected to the app (the period between the first and the last visit to the app).

Adherence rate of 56% (7.8/14 weeks) was calculated for all app users including light and heavy users. The average frequency of use during the 7.8 weeks was 4.9 days (SD 1.8).

Self-monitoring declined over the study period. At the end of 14 weeks, 27% of the users were still active on the app. According to Google Analytics, the average duration of visits was 7.5 minutes (SD 0.9). The average number of visits per user per day was 1.7 (SD 0.25), and the average number of pages per visit was 6.2 (SD 0.6).

We used the use days as a measure of compliance and divided the intervention group into light and heavy users. Light users were defined as using the app less than 3 days a week (1-40 use days), and heavy users were defined as using the app 3 or more days a week (41-98 use days). The cut-off was set at 40 days since at 40 days (almost half of the study duration) 50% of the participants were still using the app. The light and heavy users as part of the intervention group completed the 98 days (14 weeks) of the study.

The light users group included 30 participants (53% of the app group). The heavy users included 26 users (47% of the app group). At baseline, the mean weight of the light users was 77.9 kg (SD 14.8) with a BMI of 26.3 kg/m^2^ (SD 3.7); the mean weight of the heavy users was 74.4 kg (SD 15.5) with a BMI of 26.1 kg/m^2^ (SD 4.1). There were no differences at baseline between the light and heavy app users groups and the control group (*P*>.05).

The comparison between heavy and light users was a post-hoc analysis. It was not part of the main objective of the study. The main analysis depicts the comparison between intervention and control groups. The comparison between heavy and light users was added post hoc in order to illuminate the consistent trend of the beneficial effect of the app for heavy users.

### Convenience of Use

In terms of convenience, 79% of the users reported that data entry was very easy; 93% reported that use of the app was very easy. Users’ satisfaction with the app was high: 93% will make a “moderate” to “very high” recommendation to their friends to use the app. The median of the satisfaction from the app on a scale of 1-10 was 8 (25^th^ and 75^th^ percentiles were 6 and 8.5, respectively). The average score was 7.3 (SD 1.9). The app greatly affected 72.1% of the intervention group to maintain good nutrition, 69.7% reported that the app helped them lose weight, and 76.8% reported that the app encouraged them to increase their physical activity.

### Nutrition Knowledge

Table 2 shows that the score on the knowledge questionnaire did not change significantly (*P=*.16) at the end of the study in the control group but did increase significantly in the app group, from 76 (SD 7.5) to 79 (SD 8.7) at the end of the study (*P=*.04). The heavy users improved their score in the questionnaire significantly from 76 (SD 6.4) at baseline to 79 (SD 7.6) at the end of the study (*P=*.03), contrary to the light users who did not improve their knowledge significantly (*P=*.47; Table 2).

**Table 2:** Knowledge score of control and app groups and light/heavy users.

		Mean score				*P*
		Baseline	SD	End of study	SD	
**Group**						
	Control (n=29)	71	9.5	73	8.1	.16
	Web-based app (n=56)	76	7.5	79	8.7	.04
**Web-based app users**						
	Light users (n=30)	76	8.8	78	9.9	.47
	Heavy users (n=26)	76	6.4	79	7.6	.03

### Physical Activity

**Figure 4:**
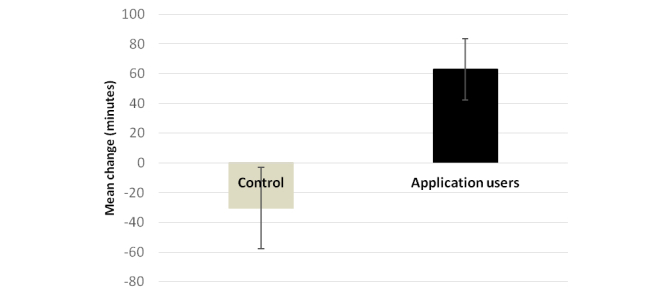
Mean change of physical activity duration in the control and app groups (P=.02).

Figure 4 presents the results of the analysis of the physical activity questionnaire. The analysis revealed that the app users significantly increased their weekly duration of physical activity compared with the control group, who decreased their weekly duration of physical activity. The mean change in the weekly duration of physical activity was 63 (SE 20.8) minutes in the app group and -30 (SE 27.5) minutes in the control group (*P=*.02; Figure 4).

Table 3 shows the percentage of participants who reached the healthy threshold of 150 minutes as recommended by the 2008 Physical Activity Guidelines for Americans [25]. At baseline, no significant difference between the control and the app groups (*P*=.42) was shown in the percentage of participants engaging in physical activity of different weekly durations.

However, at the end of the study there was a significant difference between the control group and app users. The app motivates the users to significantly increase the weekly duration of physical activity. More users increased their weekly duration of physical activity to the healthy range of more than 150 minutes a week, which may afford substantial health benefits.

**Table 3:** Percentage of participants engaging physical activity at different weekly durations.

	Study groups^a^				Web-based app users^b^			
	Control (n=29)		App users (n=56)		Light users (n=30)		Heavy users (n=26)	
	Baseline	End of study	Baseline	End of study	Baseline	End of study	Baseline	End of study
Low activity (<150 min.)	45.0%	55.0%	28.8%	17.3%	26.9%	23.1%	30.8%	11.5%
Recommended activity (150-300 min.)	30.0%	25.0%	36.5%	32.7%	42.3%	38.5%	30.8%	26.9%
High activity (>300 min.)	25.0%	20.0%	34.7%	50.0%	30.8%	38.4%	38.4%	61.6%

^a^ *P* value for difference between study groups. Baseline, *P=*.42; end of study, *P=*.01.

^b^ *P* value for difference between light and heavy users. Baseline, *P=*.64; end of study, *P=*.009.

### Diet Quality

Table 4 shows that there was no difference in the score on the diet quality questionnaire in the control group (*P=*.41). In contradistinction to the control group, the app users improved their score significantly at the end of the study from 67 (SD 9.8) to 71 (SD 7.6; *P*<.001; Table 4). No significant difference was found between light and heavy users. Both subgroups improved their quality of diet score similarly. Based on analysis of the diet quality questionnaire, the app users improved their diet quality more than the control group did.

**Table 4:** Diet quality score at baseline and at the end of the RCT.

		Mean score				*P*
		Baseline	SD	End of study	SD	
**Group**						
	Control (n=29)	61	8.9	62	10.1	.41
	Web-based app (n=56)	67	9.8	71	7.6	<.001
**Web-based app users**						
	Light users (n=30)	66	10.3	71	7.1	.017
	Heavy users (n=26)	67	9.6	72	8.2	.001

### Weight and Waist Circumference

**Figure 5:**
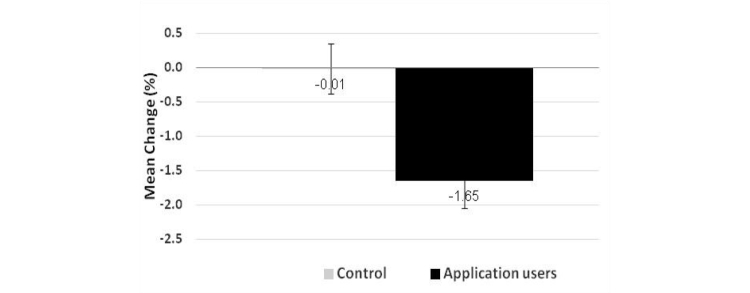
Mean change in weight (% of body weight) P=.01.

The app users lost more weight compared to the control group. At the end of the study, the mean weight and BMI change of the app group was significantly greater than that of the control group. The mean weight change of the app group was -1.44 kg (SE 0.40, 95% CI 0.1-2.5) versus -0.13 kg (SE 0.36) in the control group (*P=*.03). The mean BMI change was -0.48 kg/m^2^ (SE 0.13, 95% CI 0.05-0.84) in the app group, but only -0.03 kg/m^2^ (SE 0.12) in the control group (*P=*.03). The mean weight change presented in Figure 5 was -1.65% (SE 0.4) of the initial body weight in the app group, versus only 0.01% (SE 0.5) in the control group (*P*=.003). The mean change in weight and BMI in the heavy users was statistically significant, as opposed to the light users. Mean change in weight in heavy users was -2.1 kg (SE 0.64, *P*=.01) and in BMI -0.7 kg/m^2^ (SE 0.21, *P=*.002). Mean change in weight in the light users group was -0.84 kg (SE 0.47, *P*=.08) and BMI -0.3 kg/m^2^ (SE 0.14, *P*=.07).

No statistically significant difference was found in waist circumference between the app and control groups. At the end of study, the mean change in waist circumference was -2.31 cm (SE 0.56) in the app group and -0.9 cm (SE 0.58) in the control group (*P=*.09).

### Success Predictors

Success was defined as follows: reducing or maintaining the original weight, duration of physical activity equal to or more than 150 minutes per week, a score of more than 70 points on the nutrition and quality of diet questionnaires. The highest mean success score normalized to a range of 100% was of the app group’s 68%; 36% was the success score of the control group (*P*<.001). The heavy users received a higher mean success score (82%) than the light users (61%; *P=*.02). We did not find a difference in gender (*P*=.42) between heavy and light users. Table 5 presents the results of the linear regression model. The explained variance was 41% for the total variable parameters, and includes age, gender, weight at baseline, and frequency of app use. The key parameter for predicting success was the frequency of app use. The app frequency of use was significantly related to a higher success score (*P*<.001). The weight at baseline did not predict success, whereas being a woman did predict success.

**Table 5:** Linear regression model presenting success predictors.

Dependent variable	*R* ^2^=explained variance, %	*P* value
Age	1	.28
Weight at baseline	1	.2
Frequency of use	32	.01
Gender (female)	4	.03
Total of model variability	41	

## Discussion

### Principal Findings

In this study, we describe the development of a Web-based app for promoting healthy lifestyle and its performance evaluation using a RCT.

The app was designed as a theory-guided, technology-supported lifestyle-promoting program integrating elements pointed out in studies as important for supporting lifestyle changes. Its strengths may stem from its individually tailored approach with personal goal setting and feedback. Understanding the psychological determinants of a self-change process of altering behaviors is needed for designing effective intervention programs. The app contains features to enhance usability, feedback tailored to the user according to the DRI recommendations, a large Ministry of Health–food database, and a physical activity database.

This is one of the first studies that tested the impact of a Web-based app on lifestyle using established epidemiological methodology. The findings of the RCT indicate that the app is acceptable, effective, and can serve as a supporting tool in promoting a healthy lifestyle. These findings are strengthened by the fact that heavy users achieved better results than light users, suggesting a dose-effect relationship.

### Compliance

High compliance is a key component in the applicability of health promotion methods. The compliance to the intervention in our study was high as can be seen by the low attrition rate of 19%. This finding contrasts with an RCT by Carter et al [26], which estimated adherence to a smartphone app for weight loss compared with a website and paper diary with an attrition rate of 38%. Attrition was not distributed equally among the groups, with higher attrition in the diary and Web groups compared to the smartphone group. A review focusing specifically on Web-based interventions for weight loss found that most interventions had attrition rates greater than 20% [8]. In a systematic review of long-term weight loss trials in obese adults, attrition ranged from 30% to 60% [27]. In our study, various methods and strategies were used to support adherence to the intervention. These strategies included immediate user-tailored feedback as text messages and a graphic view of the user’s nutrition and physical activity. It seems that these strategies were effective at least in the short term in improving compliance and reducing attrition rate.

### Frequency of Use

The average frequency of the app use was found to be 2.7 (SD 1.9) days a week, and visit duration was evaluated as 8 minutes by Google Analytics. Duration of about 10 minutes per visit is sufficient to not take too much time for introducing food and physical activity, but still learn from the feedback received by the app. Brouwer [8] reported visit durations to be from less than 10-20 minutes.

In a recent literature review, Vandelanotte et al [10] reported that better outcome measures on improvement of physical activity were identified when participants visited the intervention website more than five times. However, other studies reported that only a minority of participants visited the intervention website more than once [8,28].

The frequency of self-monitoring required for successful outcomes was not clearly answered in the literature. In our study, frequency of use of 3 times per week had an added value in promoting healthy lifestyle as opposed to a lower frequency of use. Heavy users showed a trend of better outcomes and better orientation to a healthy lifestyle compared with light users.

### Adherence

The percentage of participants who adhered to the app was 56%, which is higher than described in other studies. However, it is in line with the high frequency of app use.

In the review by Kelders [29], the adherence rate of 83 interventions was 50%, and in lifestyle Web-based interventions it was only 33%. The adherence of a Web-based family intervention for overweight children was reported to be 51.1% in a 4-week study [30]. As expected, a gradual decline in the use of the app was detected. The gradual decline in adherence that was seen during the study is typical for Web-based interventions. Studies reported that 25% of the sample continues to self-monitor at the end of study [31,32]. However, some of them added in-person support. In our study, which did not include any personal support, 27% (33) of users used the app for the entire duration of the study (14 weeks). In the study by Carter et al studying adherence to a smartphone intervention [26], 12% of Web users (5 users) and 19% of smartphone users (8 users) reported for 3 months. We can conclude at this point that the adherence to the app was reasonable in our study, although increased adherence would probably improve outcomes even more.

The high rate of adherence in this study is also attributable to convenience of use, among other factors. The design of the app was oriented to enhance convenience of use. For example, the app permits one to save frequently eaten meals and thus eliminate the need for repeated searching and entry. The high percentage of users evaluating the app as easy to use is very encouraging.

### Weight and Physical Activity Change

We showed that the mean change in weekly duration of physical activity in the app group increased in our study, with more users progressing toward the recommended 150 minutes of moderate activity per week. We also found a decrease in the weekly duration of physical activity in the control group; this decrease in the control group is an interesting finding. Our expectation was that no change in any baseline measurements would occur in the control group. Some RCTs report a modest beneficial effect for the participants in control groups due to contamination. It may emphasize the importance of having a tool to encourage behavior change on a daily basis. Continuous monitoring by the app provides immediate feedback on the daily and weekly duration of physical activity and motivates for a behavior change. Without this tool (control group) physical activity duration is not consistent and depends on external factors or on the perception of the participant that the duration of physical activity is sufficient; however, reality indicates differently.

In a pilot study of smartphone-assisted behavioral obesity treatment [33], participants reported engaging in physical activity an average of 125.1 (SE 10.8) minutes per week during the first 12 weeks, and an average of 140.7 (SE 12.3) minutes per week during the second 12 weeks.

Participation in 150 minutes of moderate physical activity each week (or equivalent) is estimated to reduce the risk for ischemic heart disease by approximately 30%, the risk for diabetes by 27%, and the risk for breast and colon cancer by 21-25% [2]. Furthermore, physical activity lowers the risk for stroke, hypertension, and depression. It is a key determinant of energy expenditure and thus fundamental to energy balance and short- and long-term weight control [34]. An increased level of physical activity/exercise, regardless of body weight or weight loss, increases health [35]. Exercise offers a way to mitigate the health-damaging effects of obesity, even without weight loss [35,36]. Our findings show that users of the app increased the duration of physical activity, thus moving towards a healthier lifestyle. A larger percentage of heavy users increased the duration of physical activity to 150 minutes and above, than light users. Jing Wang [37] also reported that physical activity may increase if recording the number of minutes engaged in physical activity becomes an integral part of the self-monitoring regimen.

It is important to note that although the goal of the intervention was weight maintenance, the app users lost significantly more weight than the control group and also gained less weight, which highlights the impact of the app. Our results are consistent with studies that show a significant positive relationship between self-monitoring diet, physical activity or weight, and successful outcomes related to weight management [32].

Moreover, the same trend that more frequent app use leads to better outcomes that was seen in other comparisons was also seen regarding change in weight. Heavy users lost more weight than light users. These results are in line with other studies that claim that more frequent self-monitoring was consistently and significantly associated with weight loss compared to less frequent self-monitoring [38-40].

### Nutrition Knowledge and Diet Quality

We found that the app group received a higher score in the nutrition knowledge and diet quality questionnaires compared to the control group. Although the difference in the score between baseline and follow-up was small, it captures an increase in knowledge regarding the nutritional recommendations. This relatively small change can also be attributed to selection bias due to the increased awareness of our participants of healthy lifestyle. However, this relatively small change in knowledge score is in line with a systematic review on the impact of computer-mediated nutrition education programs for adolescents who also showed a small change in nutrition knowledge [41].

However, even promotion of small changes in eating and healthful eating can be used as preventive strategies for adults [40]. According to the World Health Organization, adequate consumption of fruits and vegetables reduces the risk for cardiovascular diseases, stomach cancer, and colorectal cancer; 1.7 million deaths (2.8%) worldwide are attributable to low fruit and vegetable consumption. Changes in specific eating patterns, such as reducing food rich in saturated fat or trans fat, can lead to a healthier diet and weight. High consumption of saturated fats and trans fatty acids is linked to heart disease. Heavy users improved their knowledge in contrast to the light users.

As the participants of the study were already inclined to a healthy lifestyle, the app was the trigger to implement what they had already known. Therefore, no difference between heavy and light users regarding diet quality was seen. However, in order to increase knowledge, the more one uses the app, the more knowledge one gains.

### Success Predictor

The success score estimates the effect of the intervention on lifestyle by integrating all measured parameters, weight at baseline, duration of physical activity, score of nutrition knowledge, and quality of diet questionnaires, into a combined score. Frequency of the app use was found to be the key factor affecting the success score. This finding supported our hypothesis that the frequency of the app use is the key factor for succeeding in maintaining or reducing weight, increasing the duration of physical activity, and improving knowledge on nutrition and diet quality.

Since studies on Web/mobile phone apps are very limited, this analysis was performed only on our app and we could not compare our findings to other studies.

### Strengths and Limitations

The app was evaluated in a randomized controlled trial. Most of the study was performed online (ie, the questionnaires and the app), with little contact between the participants and the researcher, which was limited to two meetings at the beginning and end of the study. Our findings were achieved even though there was no payment or any incentives given to the participants and face-to-face contact was limited to two meetings. Objective (Google Analytics) tools were used to evaluate the usability of the app.

However, the sample comprised predominantly adult, well-educated, white females. Therefore, the results may not be generalizable to some ethnic minority populations, adolescents, males, or less educated persons. The two study groups were unbalanced in size. The number of participants in the study group was almost twice the number in the control group. This was because it was difficult to recruit participants for the control group. The total sample was small and included 85 participants. The duration of the study was only 14 weeks. A longer duration of 12 months or more and including various biomarkers along the way is recommended for future studies. Our findings can be used as an initial 3-month testing of our intervention strategy.

### Conclusions

Analysis of the RCT results indicate that the newly developed app is effective, as reflected in high usability, increased knowledge in nutrition, increased duration of physical activity, and reduction in weight that were found in the app group only.

eBalance differs from commercially available apps in that it has taken an evidence-based approach and was guided by feedback from potential system users, contains features to enhance usability, feedback tailored to the user according to the DRI recommendations, a large Ministry of Health–food database, and physical activities database.

Testing the performance of the app in an RCT is unique and highly important for selecting the appropriate tool for public health. Evaluation of the app’s efficacy was performed by objective scientific tools. These results are promising in the app’s potential to promote healthy lifestyle, although larger and longer duration studies are needed to achieve more definitive conclusions.

## Abbreviations

ANOVA: analysis of variance
ANCOVA: analysis of covariance
DRI: Dietary Reference Intake
RCT: randomized controlled trial
